# The Global Emergence of Human Babesiosis

**DOI:** 10.3390/pathogens10111447

**Published:** 2021-11-06

**Authors:** Abhinav Kumar, Jane O’Bryan, Peter J. Krause

**Affiliations:** 1 Department of Epidemiology of Microbial Diseases, Yale School of Public Health and Yale School of Medicine, New Haven, CT 06510, USA; abhinav.kumar@yale.edu; 2 Department of Obstetrics, Gynecology & Reproductive Sciences, Yale School of Medicine, New Haven, CT 06510, USA; jane.obryan@yale.edu; 3 Frank H. Netter MD School of Medicine, Quinnipiac University, North Haven, CT 06473, USA

**Keywords:** babesiosis, *Babesia microti*, epidemiology, immunoepidemiology, case surveillance, babesiosis

## Abstract

Babesiosis is an emerging tick-borne disease caused by intraerythrocytic protozoa that are primarily transmitted by hard-bodied (ixodid) ticks and rarely through blood transfusion, perinatally, and organ transplantation. More than 100 *Babesia* species infect a wide spectrum of wild and domestic animals worldwide and six have been identified as human pathogens. *Babesia microti* is the predominant species that infects humans, is found throughout the world, and causes endemic disease in the United States and China. *Babesia venatorum* and *Babesia crassa*-like agent also cause endemic disease in China. *Babesia divergens* is the predominant species in Europe where fulminant cases have been reported sporadically. The number of *B. microti* infections has been increasing globally in recent decades. In the United States, more than 2000 cases are reported each year, although the actual number is thought to be much higher. In this review of the epidemiology of human babesiosis, we discuss epidemiologic tools used to monitor disease location and frequency; demographics and modes of transmission; the location of human babesiosis; the causative *Babesia* species in the Americas, Europe, Asia, Africa, and Australia; the primary clinical characteristics associated with each of these infections; and the increasing global health burden of this disease.

## 1. Introduction

**Figure 1:**
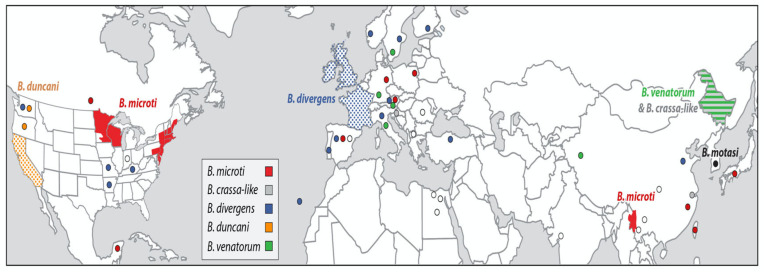
Geographic distribution of major areas of human babesiosis transmission. The map depicts the major areas where human babesiosis has been reported. Additional areas where human babesiosis has been reported but are not shown in the figure are mentioned in the text. Solid colors indicate areas where human babesiosis is endemic. Stippled areas indicate areas where babesiosis is sporadic with ≥10 cases reported. Circles depict areas where 1–10 cases have been reported. Colors distinguish the etiologic agents: *Babesia crassa*-like agent (gray), *Babesia duncani* (orange), *Babesia divergens* (blue), *Babesia microti* (red), *Babesia motasi* (black), and *Babesia venatorum* (green). White circles depict cases caused by *Babesia* spp. that were not characterized. Asymptomatic infections are omitted (adapted from The New England Journal of Medicine, Edouard Vannier, and Peter J. Krause, Human Babesiosis, 2012, 366, 2397. Copyright (2021) Massachusetts Medical Society. Reprinted with permission [1]).

Human babesiosis is caused by intraerythrocytic protozoal parasites in the phylum Apicomplexa and is transmitted by hard bodied ticks. It is rarely transmitted through red blood cell transfusion, transplacentally from mother to fetus, and through organ transplantation. Babesiosis is an emerging infection with increasing numbers of cases being reported throughout the world (Figure 1) [1,2,3,4,5,6,7,8].

More than 100 species of *Babesia* have been described that infect a wide array of wild and domestic animals [9,10]. Babesiosis is a significant problem for cattle and has had a major economic impact in several cattle producing countries. Six primary species have thus far been confirmed as human pathogens: *Babesia crassa*-like *agent, Babesia divergens, Babesia duncani, Babesia microti, Babesia motasi,* and *Babesia venatorum*. Several other genetically related pathogen substrains have been reported to infect humans, including *Babesia divergens*-like and *Babesia microti*-like pathogens (Table 1).

Human babesiosis is found primarily in the temperate zone. The predominant species is *B. microti*, which is endemic in the northeastern and northern midwestern United States and southwestern China [1,3,4,6]. *B. crassa*-like pathogen and *B. venatorum* are endemic in northeastern China [11,12]. *B. divergens* is found most commonly in Europe [2,5]. Cases of babesiosis have been sporadically reported in Australia [13], Bolivia [14], Brazil [15], Canada [16,17], the Canary Islands [18], Colombia [19], Ecuador [20], Egypt [21], India [22,23], Japan [24], Korea [25,26], Mexico [27], Mongolia [28], Mozambique [8], South Africa [29], Taiwan [30], and Turkey [31] (Table 2).

**Table 1:** First reports of *Babesia* species causing human babesiosis.

*Babesia* Species	Year Case Reported	Major Region of Transmission	Primary Vector
*Babesia microti*	1968 [32]	United States (Northeast, northern Midwest)	*I. scapularis*
*Babesia divergens*	1957 [33]	Western Europe	*I. ricinus*
*Babesia duncani*	1991 [34]	United States (Farwest)	*D. albipictus*
*Babesia venatorum* (EU1)	2003 [35]	Europe (Austria, Italy)	*I. ricinus*
		China	*I. persulcatus*
*Babesia motasi* (KO-1)	2007 [26]	South Korea	unknown
*Babesia crassa*-like agent	2018 [11]	Northeast China	*I. persulcatus*
*Genetic variants*			
*Babesia divergens*-like	1996 [36]	United States	Unknown
*Babesia microti*-like (TW1)	1997 [30]	Taiwan, Japan	Unknown

adapted from Puri et al. Frontiers in Microbiology, 2021 [37].

*Babesia* parasites were first described by Victor Babes in Romanian cattle in 1888 [38]. The first human case of babesiosis was described in 1957 by Skrabalo and Deanovic in Yugoslavia and the second in 1968 in California [32,33,39]. The causative *Babesia* species was not determined in either instance. A year later, a third babesiosis patient was reported and the causative species was identified as *B. microti.* The patient was a resident of Nantucket Island in Massachusetts where babesiosis was soon recognized as endemic [40]. Additional cases were reported in the southeastern New England mainland and from there the disease spread eastward, northward, and southward [41,42,43,44,45,46]. A primary cause of this emergence is thought to be a marked increase in the white-tailed deer population that greatly amplifies the number of vector *Ixodes scapularis* ticks. Other causes include an increase in the human population, home construction in wooded areas, increased recognition of the disease by physicians and the lay public, and improved diagnostic testing [1,39,41]. The emergence of babesiosis has lagged behind that of Lyme disease, even though it is transmitted by the same tick and is sometimes transmitted simultaneously [45,47]. Babesiosis due to *B. microti* is now endemic from Maryland to Maine and in the northern Midwestern states of Minnesota and Wisconsin. A modest number of cases of *B. duncani* have been reported on the West coast [48]. Babesiosis due to a *Babesia divergens*-like pathogen has been identified in patients in five states: Arkansas, Kentucky, Michigan, Missouri, and Washington [36,49,50,51,52].

Babesiosis should be suspected in patients who live in or travel through an endemic area or have received a blood transfusion within the previous six months and present with typical symptoms that include fever, chills, sweats, headache, and fatigue [2,53]. The disease is confirmed by identifying *Babesia*-infected red blood cells on thin blood smear or amplification of *Babesia* DNA using polymerase chain reaction (PCR) [1,2,54,55,56]. Atovaquone and azithromycin (the drug combination of choice) or clindamycin and quinine treatment are usually very effective, although prolonged illness may occur in immunocompromised hosts with a mortality rate as high as 20% [1,2,11,54,57,58].

In this review we focus on the epidemiology of human babesiosis. We will discuss epidemiologic tools used to monitor disease location and frequency, modes of transmission and demographics, the location of human babesiosis, the causative *Babesia* species in the Americas, Europe, Asia, Africa, and Australia, and the primary clinical characteristics associated with each of these infections.

**Table 2:** World-wide case distribution of human babesiosis *.

Continent/Country	Causative Agent (Number of Cases)
*Africa*	*Babesia* spp.
Egypt	*Babesia* sp. (4) [21]
Mozambique	*Babesia* sp. (2) [8]
South Africa	*Babesia* sp. (2) [29]
*Asia*	*B. crassa*-like agent, *B. divergens B. microti, B. motasi, B. venatorum*
China	*B. crassa*-like agent, *B. divergens B. microti, B. venatorum*
India	*Babesia* sp. (1) [22,23]
Japan	*B. microti* (1) [24]
Korea	*B. motasi* (2) [25,26]
Mongolia	*B. microti* (3) [28]
*Australia*	*B. microti*
New South Wales	*B. microti* (1) [13]
*Europe*	*B. crassa*-like agent, *B. divergens, B. microti, B. venatorum*
Canary Islands (Spain)	*B. divergens*-like agent (1) [18]
*North America*	*B. divergens*-like*, B. duncani*, *B. microti*
United States	*B. divergens*-like*, B. duncani*, *B. microti*
Canada	*B. microti* (1), *B. odocoilei* (2) [16,17]
Mexico	*B. microti* (4), *Babesia* spp. (3) [27,59]
*South America*	*B. microti*
Bolivia	*B. microti* (9) [14]
Brazil	*Babesia* sp. (1) [15]
Colombia	*Babesia* sp. (1), *B. bovis* (4), *B. bigemina* (2) [19]
Ecuador	*B. microti* (1) [20]

* The well-established *Babesia* spp. that cause human babesiosis in China, Europe, and the United States are listed. The *Babesia* spp. that have been identified in countries where only a few cases of human babesiosis have been identified in case reports or small case series (<10 cases) are also identified. Some causative agents have not been confirmed in larger case series so are not yet accepted as established causes of human babesiosis. *Babesia* sp. designate where a specific species was not identified.

## 2. Epidemiologic Tools

A number of methods are used to determine the frequency, location, and future emergence of infections, as part of local, state, national, and international disease tracking efforts. Case surveillance is of central importance and other methods, including case reports and case series, provide validation of surveillance data.

### 2.1. Case Surveillance

Public health officials at the local, state, and national levels collect reports of disease cases from physicians, hospitals, and laboratories. Babesiosis is one of about 120 diseases that are nationally notifiable in the United States and it was so designated in 2011. Case surveillance is of primary importance in helping the United States Centers for Disease Control and Prevention (CDC) determine the location of diseases, the number of cases of diseases at various locations, and the appropriate responses to prevent outbreaks (https://www.cdc.gov/nndss/about/index.html, accessed on 27 July 2021) [60,61,62]. Traditionally, case surveillance has been carried out through physician reporting of notifiable diseases. Recent variations on this standard approach include citizen science participation where members of the public collaborate with scientists to collect samples and data [63].

### 2.2. Case Reports and Case Series

A case report is a description of a single patient that usually includes symptoms and signs, diagnosis, and treatment. It often describes a new disease but can also describe a novel aspect of a well-known disease. Case reports include descriptions of a previously unreported disease or the presence of an emerging disease in a new location, insights into disease pathogenesis, and generation of new hypotheses or new ideas. Limitations include a lack of generalizability, inability to show cause and effect, potential for overinterpretation of the cause or outcome of disease, and a narrow focus on rare aspects of a disease [64].

A case series involves a report of a group of cases (usually more than three) that can provide information about infection transmission, risk factors for disease, diagnosis, treatment, and outcome of disease. Case series are descriptive in nature rather than hypothesis driven and are prone to selection bias and findings are often not generalizable to other populations. Despite these limitations, the publication of case reports and case series is important to raise awareness of emerging infectious diseases. Indeed, the discovery of the first human case of babesiosis was published as a case report [33]. The first reports of endemic human babesiosis were case series, describing infections due to *B. microti* [65], *B. venatorum* [12], and *B. crassa*-like agent [11].

### 2.3. Serosurveys

A serosurvey is a sera screening analysis of a group of people designed to determine the prevalence of infection. Seroprevalence provides a measurement of disease exposure and risk that is based on the antibody response of those tested [60,66,67,68]. Antibody generally can be detected about two weeks after the onset of infection and may last as little as a year or as long as a lifetime, depending on the infectious pathogen and the immune characteristics of the host. Serosurveys are one of several immunoepidemiologic tools used to improve our understanding of the epidemiology of a disease [69]. They complement case surveillance and have the advantage of capturing both asymptomatic and symptomatic infection [60]. They also inform public health officials of notifiable diseases. Thus, serosurveys are less likely to underestimate the true prevalence of infection than case finding [60,70]. One challenge of serosurveys and case surveillance methods is that antibody assays and case definitions often change over time, altering interpretation of disease trends and incidence of cases [68]. Seroprevalence surveillance may overestimate prevalence of infection if patients are repeatedly surveyed on an annual basis because antibody often persists for more than a year. Unlike case surveillance, seroprevalence does not distinguish between symptomatic and asymptomatic infection and it is symptomatic infection that better estimates the health burden of a disease.

### 2.4. Ecological Studies

#### Tick Vector and Mammalian Host Surveillance

Surveillance of tick vectors and/or reservoir hosts can provide a strong measure of risk of pathogen acquisition [3,70,71,72,73,74,75,76,77,78]. Detection methods include PCR, culture, and antibody testing. Tick vector or reservoir host surveillance only indirectly estimate the prevalence and location of human tick-borne infection but may provide a useful estimate of infection risk that complements results of human studies. In a comparative study of human and tick surveillance, incidence of Lyme disease and babesiosis were determined by reports of physicians to the Connecticut and Massachusetts Departments of Health and by reports of selected research study physicians in private practice in northeastern Connecticut and Nantucket, Massachusetts. The results of the study suggest that tick-borne surveillance can provide an early warning system for the emergence of tick-borne emerging infections [70].

### 2.5. Genomics

Genomics is an interdisciplinary branch of molecular biology that consists of the study of the structure, function, evolution, mapping, and editing of genomes. It focuses on the characterization and quantification of all the genes and their interactions that affect the function of the organism. The study of genomics has provided important new insights into the genetic basis of pathogen populations, their structure, diversity, evolution, and emergence; as well as pathogenesis, biomarkers of detection, drug resistance markers, targets for novel therapeutics, and vaccines [37,44,79,80,81,82].

### 2.6. Mathematical Modeling

Mathematical modeling is an epidemiologic tool used to study population dynamics and infectious disease transmission [83,84]. Modeling has increasingly been recognized as an important technique used to inform disease prevention and control efforts. Models may range from simple to highly complex, containing any number of parameters and variables depending on the outcome under investigation and data availability. Garner and Hamilton describe the different categories of epidemiologic models, which are classified on the basis of “treatment of variability, chance and uncertainty (deterministic or stochastic), time (continuous or discrete intervals), space (non-spatial or spatial), and the structure of the population (homogeneous or heterogeneous)” [84]. For example, in one study, laboratory and field data were integrated into a mathematical model to determine whether host coinfection with *Borrelia burgdorferi* (the agent of Lyme disease) and *B. microti* significantly increases the likelihood of *B. microti* establishment in a new previously uninfected region [45]. In another study, it was found that a model predicted that tick-borne diseases spread in a diffusion-like manner in the northeastern United States with occasional long-distance dispersal and that babesiosis spread exhibits strong dependence on Lyme disease [41].

## 3. Modes of Transmission and Demographics of Human Babesiosis

**Figure 2:**
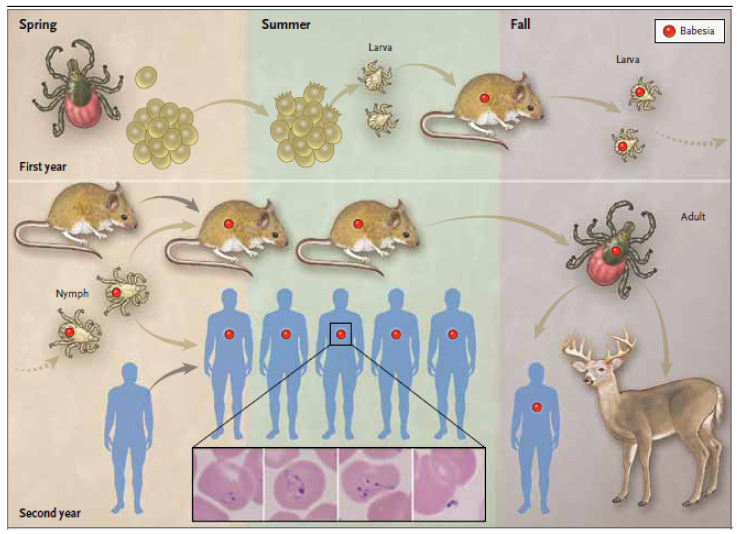
Transmission of *Babesia microti* and stages in the *Ixodes scapularis* tick vector life cycle. Female *I. scapularis* lay 2000–3000 eggs in the spring that hatch in early summer and produce larvae. Larval *I. scapularis* ticks become infected with *B. microti* when they take a blood meal from infected white-footed mice (*Peromyscus leucopus*) or other small rodent hosts in late summer. Fed larvae molt into nymphs and overwinter. During the following late spring, summer, and early autumn, infected nymphs transmit *B. microti* to uninfected mice or humans when they take a blood meal. In the autumn, nymphs molt into adults. Adult males and females preferentially feed and procreate on white-tailed deer (*Odocoileus virginianus*) but rarely on humans. The blood meal provides sufficient protein for female ticks to lay eggs. The tick life cycle is repeated when a new generation of larvae hatch from the eggs in the early spring to complete the tick life cycle. Deer do not become infected with *B. microti*. The inset panels from left to right show a *B. microti* ring form with a non-staining vacuole surrounded by cytoplasm (blue) and two small nuclei (purple), an amoeboid form, a tetrad form (also referred to as a Maltese cross), and an extracellular form (adapted from The New England Journal of Medicine, Edouard Vannier, and Peter J. Krause, Human Babesiosis, 2012, 366, 2397. Copyright (2021) Massachusetts Medical Society. Reprinted with permission [1]).

*Babesia* spp. perpetuate in nature through a tick-vector and mammalian-host cycle [39]. Vectors and hosts differ for each species of *Babesia* and vary geographically but the basic tick–host transmission cycle is similar for all [1]. The life cycle for *B. microti* is shown in Figure 2 with *I. scapularis* as the tick vector but other tick species serve as vectors for other *Babesia* spp. (Table 1). *Peromyscus leucopus* is the primary reservoir for *B. microti* but other small mammals, such as shrews and chipmunks, can also serve as reservoir hosts for *B. microti* and other *Babesia* spp. [39,85]. Similarly, deer and other large mammals are favored hosts for adult ixodid ticks. In some *Babesia* spp., such as *B. divergens*, this transstadial transmission is supplemented by transovarial transmission from mother to egg [7,86]. Deer markedly amplify tick numbers and are largely responsible for the emergence of *Babesia* and other tick-borne infections over the last three decades in the Northeast and northern Midwest regions of the United States [39,70].

*B. microti* are primarily transmitted by *I. scapularis* ticks and rarely through blood transfusion, organ donation, and transplacentally [1,39,87,88,89]. Babesiosis has been one of the leading causes of transfusion transmitted infection in the United States [87,90]. More than 250 cases have been reported and approximately one-fifth of these cases have been fatal [87]. Blood donor screening for *B. microti* is an effective preventative measure [91,92]. In 2020, the United States Food and Drug Administration recommended donor screening in 14 *B. microti* endemic states and Washington D.C. using approved PCR technologies. Initial data indicate that the numbers of transfusion-transmitted cases has markedly decreased.

Ten cases of congenital babesiosis due to *B. microti* have been described [88,93]. Strong supportive evidence indicates that these cases were not due to transfusion or tick transmission and definitive evidence was available for several cases. Congenital babesia infection is not always severe in neonates and there have been no fatalities. *B. microti* infection also has been reported in two kidney transplant recipients who received kidneys from a single infected kidney donor [89].

**Figure 3:**
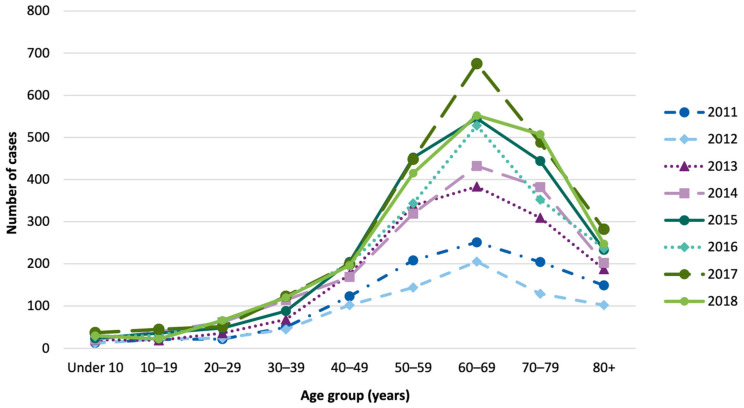
Babesiosis cases by age in the United States. Babesiosis cases reported by age to the Centers for Disease Control and Prevention, United States between 2011 and 2018 are shown. The low numbers of cases in children is due to the mild clinical symptoms resulting from *B. microti* infection rather than exposure to the infection. Almost half of children are asymptomatically infected compared to about a fifth of adults. Thus, *B. microti*-infected children are not diagnosed as frequently as adults (adapted from the Centers for Disease Control and Prevention. Notifiable Diseases and Mortality Tables. MMWR Morb Mortal Wkly Rep 2016, 65(3) [95]).

The peak age of reported human *B. microti* cases in the United States is between 60 and 70 years of age (Figure 3). Very few cases are reported in children. In contrast, serosurveys show that children are infected as frequently as adults. Children have much milder disease and the diagnosis is more often missed in children. Indeed, about 40% of children are asymptomatically infected compared with about 20% of adults [60,94]. Babesiosis is reported more frequently in males than females, presumably because they are more often exposed to tick-infested areas. Lawn maintenance workers and those with occupational exposure to ticks are at greater risk of tick-borne diseases than the general population.

## 4. Human Babesiosis in the Americas

### 4.1. Overview

The first case of babesiosis in the United States was described in 1968 in a California resident, although the species was not identified [32]. Two years later, a case of *B. microti* was described in Nantucket, Massachusetts [40]. Subsequent reports on Nantucket established this island as the first babesiosis endemic site. The disease became known as Nantucket fever [65]. Subsequently, cases were reported on Cape Cod, Massachusetts, and the New England mainland. The reports of babesiosis subsequently broadened from southern New England to include endemic areas from Delaware to Maine [41,42,44,81,82,96,97]. Recent genomic studies have established that the initial source of *B. microti* was not from Nantucket but rather from the mainland in southeastern New England [44,81,82]. A similar emergence of babesiosis in Wisconsin and Minnesota is ongoing [81,98].

**Figure 4:**
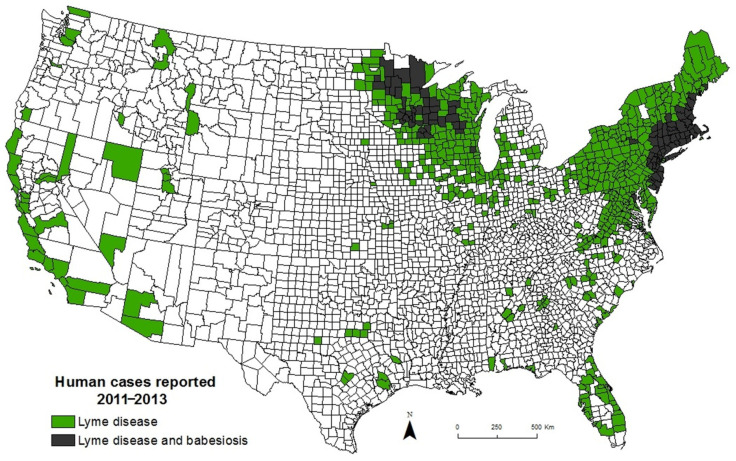
Human babesiosis occurs within Lyme disease endemic areas in the United States. Lyme disease and human babesiosis have been nationally notifiable conditions since 1991 and 2011, respectively. The names of counties that reported cases of Lyme disease and/or babesiosis from 2011 to 2013 were obtained from the Centers for Disease Control and Prevention. Counties with three or more cases of Lyme disease but fewer than three cases of babesiosis are depicted in green. Counties with three or more cases of Lyme disease and three or more cases of babesiosis are depicted in gray. No county reported three or more cases of babesiosis but fewer than three cases of Lyme disease (adapted from Diuk-Wasser M, Vannier E, Krause PJ. Coinfection by *Ixodes* tick-borne pathogens: Ecological, epidemiological, and clinical consequences. Trends Parasitol 2016, 32, 30–42 [43]).

The emergence of babesiosis in the Northeast is thought to be due to several factors, including increased recognition of babesiosis by health care workers and the general public, an increase in the human population, construction of homes near wooded areas where ticks abound, and a marked increase in the deer population [41,71,96,97,99,100,101]. In the late 19th century, the number of deer in the United States had decreased to an estimated 300,000 due to hunting and the loss of forest habitat for farmland. Deer sightings in New England at that time were mentioned in local newspapers [39]. As farming moved to the Midwest and hunting declined, the deer population steadily increased to about 30 million in 2017. An increase in the white-tailed deer population has been accompanied by a marked increase in the *I. scapularis* population and a concomitant increase in the number of cases of Lyme disease, while removal of deer from specific locations has greatly diminished the number of ticks and cases of Lyme disease [39,102,103,104]. Interestingly, Lyme disease has spread more widely than babesiosis, in part because *B. microti* is less efficiently transmitted than *B. burgdorferi* [41,45]. There are large areas of the Northeast and northern Midwest where Lyme disease is endemic but babesiosis is not. There are no areas where babesiosis is reported in the absence of Lyme disease (Figure 4). Laboratory studies suggest that Lyme disease/babesiosis coinfection enhances the transmission of babesiosis and it has been hypothesized that the establishment of Lyme disease in an area is a prerequisite for the establishment of babesiosis [43,45]. Furthermore, birds can serve as hosts for *B. burgdorferi* but not *B. microti*. Larval ticks may attach and feed on *B. burgdorferi*-infected birds and be deposited hundreds of miles away where they can then establish a new site of infection. Both *B. burgdorferi* and *B. microti* can spread from one infected colony of mice to an adjacent colony but spread in this case is much slower than with birds [39,41].

### 4.2. United States

#### 4.2.1. *Babesia microti* Infection

Currently, 14 states account for the vast majority of babesiosis cases in the United States and most are due to *B. microti*. These states include Connecticut, Delaware, Maine, Maryland, Massachusetts, Minnesota, New Hampshire, New Jersey, New York, Pennsylvania, Rhode Island, Virginia, Vermont, and Wisconsin [46,95]. Geographic modeling suggests that babesiosis will continue to emerge in the United States. The areas presently endemic for babesiosis and Lyme disease are expanding toward each other from the Northeast and Midwest. It has been postulated that a continuous endemic band of these two diseases may someday extend from Minnesota to the East coast. Lyme disease also is expanding into southeastern Canada and this is thought to be due, at least in part, to climate change [105,106].

Clinical manifestations of *B. microti* illness vary from subclinical illness to fulminating disease resulting in death [1,39,60,100,107]. Fever typically develops after a gradual onset of malaise, anorexia, and fatigue and may reach 40 °C (104 °F) [1,96,100,108]. Other common symptoms include chills, sweats, myalgia, arthralgia, nausea, and vomiting. Physical examination of *B. microti*-infected patients reveals fever and occasionally mild splenomegaly, hepatomegaly, or both. Abnormal laboratory findings include hemolytic anemia, elevated renal function and liver enzyme levels, and thrombocytopenia [58,96,100]. The illness usually lasts for a week or two but occasionally several months, with prolonged recovery taking up to 18 months [58,109]. Persistent parasitemia and clinical and microbiological relapse have been described for as long as 27 months after the initial episode, due in part to the development of antibiotic resistance [58,81,110,111,112]. Severe *B. microti* illness requiring hospital admission is common in patients with splenectomy, malignancy, HIV infection, hemoglobinopathy, chronic heart, lung, or liver disease, organ transplantation, acquisition of babesiosis through blood transfusion, and in newborn infants and the elderly [1,2,4]. Complications include severe hemolytic anemia, congestive heart failure, acute respiratory distress syndrome, disseminated intravascular coagulopathy (DIC), renal failure, coma, and shock [54,100,107]. 

#### 4.2.2. *Babesia duncani* Infection

In 1991, a 41-year-old resident of Washington State presented with viral-like symptoms and was diagnosed with babesiosis. The causative pathogen was propagated in hamsters and was found to be morphologically similar but genetically and antigenically distinct from *B. microti* [34]. The organism was named WA-1. Eight additional cases of babesiosis with recovery of the same causative *Babesia* pathogen were subsequently reported in California and Washington states. The *Babesia* were found to be morphologically, ultrastructurally, and genetically indistinguishable from one another and were subsequently named *Babesia duncani* [48]. Two additional cases have been described in California and Oregon, respectively. The primary vector is *Dermacentor albipictus* [113]. Limited data suggests that the clinical manifestations of these cases are similar to those of *B. microti*. There is a marked difference in disease severity in hamster and C3H/Hen mouse models, however, as *B. duncani* causes fatal illness while *B. microti* causes mild or asymptomatic infection [114,115].

#### 4.2.3. *Babesia divergens*-Like Infection

In 1996, Herwaldt and colleagues described a fatal case of babesiosis in a 73-year-old asplenic resident of Missouri who was infected with a *Babesia* that shared morphologic, antigenic, and genetic characteristics with *B. divergens*. The patient had previous exposure to cattle. The pathogen was named MO1 [36]. Four similar cases of *B. divergens*-like organisms have subsequently been described, none with exposure to cattle: (i) a 56 year old asplenic male resident of Kentucky who survived [49]; (ii) an 82-year-old asplenic male resident of Washington State with hypertension and secondary renal insufficiency who survived [50]; (iii) an 81-year-old asplenic Arkansas resident with diabetes, coronary artery disease, chronic obstructive pulmonary disease, a history of mitral valve replacement, hypertension, and GI bleeding, who died [51], and (iv) a 60-year old asplenic female resident of Michigan who developed multiple organ failure but survived [52]. These cases were similar to those of *B. divergens* cases from Europe, where almost all have occurred in asplenic individuals and many have died (see below).

#### 4.2.4. Coinfection

Several different human pathogens cycle between *I. scapularis* ticks and mammalian reservoir hosts in the United States, including *Anaplasma phagocytophilum*, *Babesia microti*, *Borrelia burgdorferi*, *Borrelia mayonii*, *Borrelia miyamotoi*, deer tick virus (Powassan virus), and *Ehrlichia muris*-like organism [116]. These pathogens differ in their geographic range within the Northeast and northern Midwest. In areas where two or more pathogens are enzootic, simultaneous infection (coinfection) may occur. In the first case series of coinfection, the frequency and clinical outcome of Lyme disease and babesiosis alone were compared with those of Lyme disease and babesiosis coinfection [47]. Eleven percent of Lyme disease patients experienced coinfection while 72% of babesiosis patients had coinfection. This was expected because of the much larger number of Lyme disease patients compared with babesiosis patients. Lyme disease patients had a greater number of symptoms for longer duration if they were coinfected with *B. microti* [43,47]. The percentage of patients experiencing coinfection varies geographically and depends on the relative incidence of Lyme disease and babesiosis.

In addition to exacerbating human disease severity, *B. microti-B. burgdorferi* coinfection appears to increase *Babesia* parasitemia in the natural mouse reservoir, leading to greater transmission of *B. microti* from mouse reservoir to tick vector [45]. This enhancement of otherwise less transmissible *B. microti* may help explain why babesiosis has emerged more slowly than *B. burgdorferi* and is only found in areas of the United States where Lyme disease is endemic. Additional data suggests that coinfection provides a survival advantage for both *B. microti* and *B. burgdorferi* [43].

### 4.3. Canada

The first case of babesiosis in a Canadian resident was reported in 1999 [117]. The patient had traveled to Nantucket, Massachusetts six weeks prior to disease onset, indicating that the *Babesia* sp. identified on blood smear may not have been acquired indigenously. A second case of babesiosis was reported in 2001 in a 53-year-old Canadian resident who most likely acquired infection through blood transfusion from an asymptomatic *B. microti* positive donor [16]. The donor was thought to have acquired his infection in Cape Cod, Massachusetts. The first definitive case of locally acquired babesiosis in Canada was reported in a seven-year-old asplenic resident of Manitoba [17]. The child had not traveled outside Manitoba and never had a blood transfusion. *Babesia* were demonstrated on blood smear and *B. microti* was identified as the causative *Babesia* sp. by PCR. *I. scapularis* ticks infected with *B. microti* have been found in six different localities in Manitoba. Recently, two cases of *Babesia odocoilei* have been described with typical symptoms of babesiosis and positive PCR testing [118].

### 4.4. Mexico

A *Babesia* serosurvey was performed in Las Margaritas, Mexico in 1976. The sera of one third of 101 study subjects reacted against a dog *Babesia* antigen (*Babesia canis*) [59]. Three seropositive residents were found to be infected with *Babesia* when their blood was injected into splenectomized hamsters and *Babesia* were isolated from the hamsters. The *Babesia* species could not be identified. Four decades later, babesiosis due to *B. microti* was described in Yucatan State, Mexico [27]. The four patients ranged in age from 8 to 14 and lived in close proximity to each other in a rural area of eastern Yucatan. All subjects had tick bites or lived in tick-infested areas. All experienced mild to moderate illness with fever and three also experienced fatigue, arthralgia, and myalgia. The diagnosis was confirmed and the infecting species identified by amplification of *B. microti* DNA using PCR. All were given chloroquine and had a full recovery despite the fact that chloroquine is not effective for the treatment of human babesiosis.

### 4.5. South America

Two cases suggestive of babesiosis were reported in 2003 in South America. One was a 37-year-old resident of Puerto Berrio, Colombia who had fever, chills, sweats, weakness, and bone aches. *Babesia* parasites were identified on thin blood smear. A PCR was not performed but the patient had an antibody titer of 1:64 against *Babesia bovis* antigen [19]. The second case was an asymptomatic 2-year-old from Brazil with hepatoblastoma who had a positive blood smear for *Babesia* [15]. No *Babesia* PCR or antibody testing were performed.

In a survey of 300 residents of two rural towns (Turbo and Necocli) in Colombia where cattle ranching is an important industry, four subjects tested positive for *B. bovis* by PCR, including two who were blood smear positive [119]. Another two residents tested positive for *B. bigemina* by PCR, including one whose blood smear was positive. Three of these subjects were symptomatic with fever and/or headache and three were asymptomatic. Human babesiosis due to *B. bovis* and *B. bigemina* had not previously been described.

Nine cases of asymptomatic *B. microti* infection were discovered among 271 healthy residents of two rural towns in southeastern Bolivia [14]. All nine cases had *Babesia* identified on thin blood smear and further characterized as *B. microti* by PCR and molecular sequencing. All cases were seropositive when tested with a standard *B. microti* immunofluorescence antibody (IFA) assay.

A 72-year-old patient from Ecuador with chronic abdominal pain moved to Chicago and two months later developed fever, chills, headaches, myalgia, dry cough, nausea, vomiting, and diarrhea. He was admitted to the hospital and diagnosed with malaria based on his country of origin, symptoms, a positive blood smear showing intraerythrocytic ring forms (parasitemia 0.5%), and positive *P. falciparum* IgG antibody. A blood sample sent to the CDC was positive for *B. microti* by PCR. His infection resolved on atovaquone and proguanil [20].

In summary, there is evidence of human *B. microti* and other *Babesia* spp. infection in South America. Additional studies are necessary to better define the scope of the problem there, including confirmation of other *Babesia* species causing human infection.

## 5. Human Babesiosis in Europe

### 5.1. Overview

The first documented case of human babesiosis anywhere in the world was reported in the former Yugoslavia in 1957 [33]. The affected patient was a splenectomized farmer who succumbed to severe hemolytic anemia. The parasite species was never determined but *B. bovis* was found in the cattle he tended [120]. Since then, more than 50 cases of babesiosis have been reported on the European continent [1,5,121,122,123]. The predominant pathogen in Europe is *B. divergens,* however, *B. microti* and *B. venatorum* have been identified in a small number of cases [35,124,125]. A case of *B. divergens*-like infection has been reported in the Canary Islands (Spain) [8]. A comprehensive review of human babesiosis in Europe by Hildebrandt et al. (2021) documented a total of 51 autochthonous cases, with 35 attributed to *B. divergens*, 11 to *B. microti*, and 5 to *B. venatorum* [2]. Epidemiologic surveys have indicated widespread distribution of *B. divergens* and its associated tick vector, *Ixodes ricinus,* throughout Europe [126]. Recent seroprevalence reports suggest a much higher clinical incidence than has been described in the extant literature to date [127]. Quantitation of true babesiosis incidence across Europe remains a challenge because symptoms often manifest non-specifically, immunocompetent individuals are frequently asymptomatic, and babesiosis is not a notifiable disease in many countries [128].

### 5.2. Babesia divergens

*B. divergens* is the primary causative agent of human babesiosis in Europe and is endemic in the European cattle population. Gray (2006) described the ecological landscapes of countries with the highest incidence of bovine babesiosis as having significant tick populations in “rough open hill-land or damp low-lying meadows” and “where woodland frequently abuts cattle pasture” [129]. Over half of the cases of European babesiosis have been reported in France and the British Isles, with at least 10 other countries represented in single case reports [31,122,130,131,132,133]. Prevalence of babesiosis is reportedly increasing, and the European Center for Disease Prevention and Control have identified several factors driving this trend: landscape modifications affecting tick populations, deer population growth, human activity in infested areas, and dissemination of pathogens through cattle movement (https://www.ecdc.europa.eu/en/all-topics-z/babesiosis/facts-about-babesiosis, accessed on 27 July 2021). Disease emergence at increasingly northern latitudes in Europe has been recently observed. Mysterud and colleagues analyzed longitudinal tickborne disease incidence data from Norway and found that this emergence is linked to tick vector distribution [134]. *I. ricinus* is the primary vector of *B. divergens* and is widely distributed across Europe [135]. Primary host species include domesticated cattle, [126], roe deer, and other cervids (e.g., moose, red deer, reindeer, sika deer) [136].

*B. divergens* infections are characterized by fulminant disease and all but a few cases have been reported in asplenic patients [5,123,128]. Factors that predispose patients to severe disease include the extremes of age and other causes of immunocompromised clinical status [122,137]. After an incubation period of 1–3 weeks, *B. divergens* symptoms generally have a rapid progression with high fever, chills, sweats, headache, myalgia, hemolytic anemia, and hemoglobinuria [5]. Mortality associated with *B. divergens* infection, often due to multiorgan failure, was previously estimated to be as high as 42% but is improving. Better outcomes are thought to result primarily from more aggressive therapy, including intravenous antibiotics and the early use of exchange transfusion [5]. Two recent publications have challenged this “classic description of babesiosis in Europe.” Martinot et al. described two exceptional cases of severe babesiosis in healthy, young, immunocompetent patients in France, and Gonzalez described a similar case in Spain [123,138].

### 5.3. Babesia venatorum

*B. venatorum* is an emerging public health concern in Europe due to its widespread zoonotic presence [136]. *B. venatorum*, formerly referred to as *Babesia* sp. EU1, is closely related to *B. divergens* and *B. odocoilei* [35,139]. Wild hosts include roe deer and moose [136]. The parasite has also been detected in captive reindeer and domesticated sheep [75,140,141,142]. The *I. ricinus* tick acts as both vector and reservoir. Cases in humans have thus far been reported in Austria, Germany, and Italy [35,124]. Case reports have described disease manifestations ranging from mild to moderately severe, which resolve with antimicrobial therapy, even in the setting of asplenia and lymphoma. The clinical presentation of *B. venatorum* infection is generally less severe compared to that of *B. divergens* [5].

### 5.4. Babesia microti

Cases of *B. microti* infection have been reported from Austria, Germany, Italy, Poland, Spain, and Switzerland [35,125,143,144,145,146]. The first evidence of human *B. microti* infection in Europe was a report of seropositive residents in Switzerland in 2002 [147]. A number of serosurveys have shown a wide range of *B. microti* seropositivity depending on the location and study population (e.g., general public, forest workers, Lyme disease coinfected subjects). *B. microti* seropositivity has ranged from 0.5% to 32% in study populations in Belgium, France, Germany, Italy, Poland, Sweden, and Switzerland [139,147,148,149,150,151,152,153] Furthermore, Hunfeld et al. (2002) reported that IgG seroprevalence rates were higher for *B. microti* (9.3%) than for *B. divergens* (4.9%) among patients exposed to ticks in Germany. [139] These seroprevalence data indicate that there is more human *B. microti* infection in Europe than currently identified.

### 5.5. Babesia crassa-Like Agent

*Babesia crassa* is a relatively uncommon *Babesia* species with documented infection in sheep in Iran and Turkey [154]. A single case of *B. crassa*-like infection has been reported in Europe in Slovenia [155]. The patient in question was asplenic and recovered after standard antibiotic treatment. Cases subsequently have been described in China.

## 6. Babesiosis in Asia

### 6.1. Overview

Several countries in Asia have reported human cases of babesiosis, including China, India, Japan, Korea, and Mongolia. In addition to previously documented human *Babesia* pathogens, several new *Babesia* species have been found to infect humans. As with any single case report of a novel *Babesia* species or report of a known *Babesia* sp. in a new region, identification of additional cases and pathogen isolation from local tick vectors and mammalian hosts will help confirm original findings [22,156]. The increasing interest and reports of human babesiosis in Asia are likely to reveal additional species and new areas of endemicity.

### 6.2. China

#### 6.2.1. Human Infection

Outside the United States, the greatest number of human babesiosis cases are reported in China. China is the only country, other than the United States, where babesiosis has been shown to be endemic. Babesiosis in China is considered an emerging public health threat [3,6,157,158]. Among the human *Babesia* spp. identified to date, four (*B. microti*, *B. divergens*, *B*. *venatorum*, and *B. crassa*-like agent) have been confirmed to cause human infections in China [11,35,159,160,161,162,163,164,165]. Studies in western China more than a decade before the first official report of human babesiosis in Yugoslavia described *Babesia*-like intraerythrocytic organisms associated with febrile illness that may have been *Babesia* [6].

#### 6.2.2. *Babesia venatorum*

*B*. *venatorum* was found to cause infection over a two year study period in Heilongjiang province in northeastern China, indicating endemic transmission. The majority of tick-borne cases in China are found in this province. Jiang et al. screened 2912 individuals for microscopic, PCR, or animal inoculation evidence of *Babesia* spp. infection in patients who reported a recent tick-bite and who sought hospital care between 2011 and 2014. Results showed that 48 (0.16%) of these patients had *B. venatorum* infection [12]. The *B. venatorum* 18S RNA gene sequences from all 48 patients were identical and differed from European *B. venatorum* parasite isolates by only two nucleotides. These data suggested a common origin of *B. venatorum* spp. in parasites circulating in northeastern China and Europe. Only five cases of *B. venatorum* had previously been identified, four of which were in Europe and one in a child in China [6,35,124,164,166,167].

#### 6.2.3. *Babesia crassa*-Like Agent

A similar study led to the discovery of *B. crassa*-like pathogen as another causative agent of endemic human babesiosis in China. Between May 2015 and July 2016, Jia et al. screened 1125 residents of Heilongjiang Province for evidence of *Babesia* spp. infection who experienced fever and recent tick-bites. Of these participants, 5.0% (58/1125) demonstrated the presence of a novel *B. crassa*-like species in their blood, based on species-specific PCR testing and nucleotide sequencing [11]. *B. crassa*-like parasites were visualized on thin blood smears and showed ring, ameboid (<3 μm in size), and tetrad forms. The authors characterized the severity of disease manifestations as mild to moderate. Interestingly, 7.5% of healthy, asymptomatic residents of the area tested positive for *B. crassa*-like infection, suggesting that many human babesiosis cases due to *B. crassa*-like pathogen go undetected in China [11].

DNA samples also were collected from 1732 adult ticks from May to July 2014 from the same study area. Nine *I. persulcatus* and *Haemaphysalis concinna* ticks showed the presence of *B. crassa*-like species. Blood samples collected from 5 of 1125 sheep contained *B. crassa*-like DNA [11]. The *B. crassa*-like species is phylogenetically related to *B. crassa*, a large *Babesia* parasite of sheep in Turkey and Iran [154,168]. The near full length *B. crassa*-like 18S rRNA gene sequences showed 96.7% and 97.7% sequence similarities with the *B. crassa* sequences, respectively, from sheep in those countries [11].

#### 6.2.4. *Babesia microti*

*B. microti* is another important *Babesia* sp. that causes human babesiosis in China [158]. Phylogenetic analyses based on the sequences from the 18S rRNA gene have revealed that *B. microti* from China are phylogenetically similar to those from Japan and Switzerland [6]. Clinical cases attributed to *B. microti* have been reported sporadically from Zhejiang, Yunnan, and Guangxi provinces [158,160]. Accurate diagnosis of clinical babesiosis is a challenge where *B. microti* babesiosis and malaria coexist in the same area in southwestern China, specifically Yunnan Province along the China–Myanmar border. The first reported cases of co-infections of *B. microti* and *Plasmodium* spp. were discovered there in 2012–2013 [165]. *B. microti*, *P. falciparum*, *P. vivax*, and *P. malariae* infections were identified among 449 febrile patients. Eight patients (1.8%) had infection with *B. microti* alone while 10 (2.2%) were co-infected with *B. microti* and either *P. falciparum* or *P. vivax* [165]. These results clearly illustrate a possible hidden clinical burden of *B. microti* in malaria endemic areas where babesiosis is not known to exist. Furthermore, patients experiencing febrile illness with intraerythrocytic parasites on blood smear may be misdiagnosed as having malaria when they actually have babesiosis.

*B. microti* has been shown to be transmitted by blood transfusion in the United States and Japan. Very limited data is available on the transmission risk of *B. microti* in Chinese blood donors. A single case of transfusion-associated babesiosis in China has been reported. *B. microti* was identified as the causative agent [169]. Large scale molecular and serological surveys to assess *Babesia* spp. risk among random blood donors in China are not yet available. A 2016 pilot serosurvey of blood donors in Heilongjiang Province revealed that 13 of 1000 (1.3%) donors had antibodies against *B. microti* parasites by the immunofluorescence antibody assay [161]. This *B. microti* antibody positivity rate is comparable to rates observed in blood donors in endemic areas in the northeastern United States [170]. These results provide further evidence that the prevalence of *B. microti* transmission in China may be significantly higher than currently realized and might be comparable to prevalence in the United States.

#### 6.2.5. *Babesia divergens*

In recent years, laboratory screenings of probable cases of babesiosis in patients presenting to Chinese hospitals with recent tick bites have yielded surprising findings that are suggestive of the presence of novel *Babesia* spp. The first case of *B. divergens* (cattle *Babesia* sp.) infection in China was identified in a patient in 2011. The 18S rRNA gene sequence from this individual had 98.4% similarity with the gene of *B. divergens* in Switzerland [162]. A subsequent study in Gansu Province of 754 patients who visited a hospital for a tick bite between April and March 2016 showed that 10 patients (1.3%) had *B. divergens* infections, based on positive PCR tests [163]. *B. divergens* sequences from this study site were 99.9% identical to sequences of *B. divergens* from Europe. Interestingly, *B. divergens* infection has never been identified in cattle in China, possibly indicating a different reservoir host for this *Babesia* sp. Another salient feature of this study was that all 10 *B. divergens* infected patients were immunocompetent and only two had clinical symptoms at the time of sample collection.

#### 6.2.6. Tick-Vectors and Animal Hosts of *Babesia* spp. in China

Several entomological and molecular studies have allowed quantitation of tick-vector and reservoir host infection rates, as well as geographic distribution of *Babesia* spp. in China. Fang et al. (2015) published a comprehensive overview of tick-borne infections in tick vectors, animal hosts, and humans [3]. The authors reported a total of 33 emerging tick-borne agents that have been identified in mainland China, including 11 species of *Babesia*. Their analyses showed that transmission of *Babesia* spp. is associated with 13 tick species. Although more prevalent in the northeastern regions, *Babesia* spp. were distributed throughout China.

Among *Babesia* spp. that infect humans, *B. venatorum* has been reported in *I. persulcatus* ticks from northeastern China [3]. *B. crassa*-like agent has been detected in *I. persulcatus* and *H. concinna* ticks from sheep in the same area in Heilngjiang Province [11]. *B. microti* has been identified over a broad expanse of China, including, (i) *I. persulcatus* and *H. concinna* ticks and striped field mice and reed voles in Heilongjiang Province, (ii) *H. longicornis* ticks on dogs from Henan Province, and (iii) rodents from Fujian, Zhejiang, Henan, and Heliongjiang provinces. *B. divergens* has been detected in *I. persculcatus*, *H. concinna,* and *Haemaphysalis japonica* ticks and in striped field mice in Heilongjiang Province. *Babesia* spp. that have not been shown to cause human infection in China include *B. ovis*, *B. major*, *B. ovata*, *B. orientalis*, *B. motasi*, *B. caballi*, *Babesia* sp. Kashi, and *Babesia* sp. Xinjiang [3]. More recently, Xia et al. have performed genotyping of *Babesia* spp. in a total of 2380 *I. persulcatus* and *H. concinna* ticks in a narrow forested area at 30 sampling points in northeastern China based on the 18S rRNA gene sequences [76]. Results showed that 23 (0.97%) of *I. persulcatus* ticks tested positive for five *Babesia* spp.—*B. bigemina*, *B. divergens*, *B. microti*, *B. venatorum* and one novel strain HLJ-80. Thirteen *H. concinna* ticks were positive for the following *Babesia* spp.—*B. bigemina*, *B. divergens*, three genetic variant forms of HLJ-874, and eight other *Babesia* variants represented by HLJ 242, which were similar to *B. crassa* [76]. The authors concluded that each site contained 5–6 different *Babesia* spp., several of which are capable of infecting humans. Additionally, Kobi-type and Otsu-type *B. microti* have been detected in wild rodents in Yunnan Province [171]. Overall, the presence of a number of *Babesia* spp. and their genetic variants infecting tick vectors and animal hosts indicate a high *Babesia* transmission risk to humans living in different parts of China.

### 6.3. India

A single case of human babesiosis was described in a resident of north central India in 2005. The diagnosis was confirmed by identification of *Babesia* on thin blood smear but the species was not identified. Antigen tests for *Plasmodia* were negative [22,23].

### 6.4. Japan

In 1980, Shiota et al. documented the presence of *B. microti* parasites in blood films collected from Japanese field mice [172]. The only autochthonous case of human babesiosis that has been reported from Japan was in a patient who acquired infection through blood transfusion during admission to Kobi University Hospital, Hyogo Prefecture in 1999 [24]. *B. microti* parasites were confirmed by blood smear microscopy and PCR analysis. The parasite isolated from the index patient’s blood sample and from a blood sample inoculated and propagated in SCID mice were identified as a *B. microti*-like parasite, which had a 99.2% sequence homology with the *B. microti* reference strain from the US [24]. Although a blood sample from the implicated asymptomatic donor collected eight months after the index donation was negative for *B. microti* parasites by blood smear microscopy and PCR analysis, inoculation into SCID mice allowed detection of *B. microti* parasites that had sequence identity with the parasite isolate from the blood recipient [173]. *B. microti*-parasites exhibiting a similar genotype as the index patient and the asymptomatic blood donor were also isolated from a field mouse near the donor’s residence, indicating enzootic and zoonotic transmission of *B. microti* in the area [173].

Molecular surveillance studies in the presumed *I. persulcatus* tick vector and field mouse reservoir host have demonstrated the presence of *Babesia* spp. throughout Japan with a potential for human transmission [173]. A field survey in Hokkaido Prefecture revealed the presence of *B. divergens* (Asia lineage) parasites in *I. persulcatus.* The presence of *B. microti* (United States lineage) and *B. venatorum* (strain Et65) were also noted in the same tick species [174]. Sika deer (*Cervus nippon*) were shown to carry *B. divergens* parasites in different Japanese prefectures [175]. In a more recent study, hard ticks belonging to the genera Ixodes and Haemaphysalis collected from sika deer in Hokkaido were found to harbor DNA for *B. microti*, *B. microti* Hobetsu, and *B. divergens*-like (Bab-SD) parasites [176]. Together, these studies suggest a wide-spread presence of *Babesia* spp. in tick vectors, mouse reservoir hosts, and humans in Japan.

### 6.5. Korea

Two cases of human babesiosis have been documented in Korea. In the first case, a blood sample from a patient contained paired pyriform and ring forms of *Babesia* parasites. The parasite isolate was named *Babesia* sp. KO1 and was found to be genetically related to sheep *Babesia* in China [26]. In the second case, a parasite isolate from a symptomatic patient was found to be closely related to *B. motasi*, a sheep parasite. Tick samples collected nearby the patient’s residence demonstrated the presence of *B. microti* and *B. motasi* DNA (98% homology) [25]. Limited data is available for the tick-vectors and reservoir hosts of *Babesia* spp. in Korea. In one study, *B. microti* parasites (United States type) were detected by PCR in blood samples from wild animals in Gangwon-do Province [177]. In another study, *B. microti* (United States type) DNA was detected in blood samples from *Apodemus agrarius* (striped field mouse) but was absent from the other small mammals that were screened [28].

### 6.6. Mongolia

A survey of 100 asymptomatic farmers in Selenge province, Mongolia revealed that 7% had *B. microti* antibody and 3% had amplifiable *B. microti* DNA in their blood [28]. In a more recent study, a third of 63 questing *I. persulcatus* ticks were found to be infected with *B. microti* (United States type) in Selenge province in Mongolia [178].

## 7. Babesiosis in Africa

There have been very few cases of human babesiosis reported on the African continent to date. Human babesiosis caused by unknown species have been described in Egypt and Mozambique [8,21,121]. Two cases of babesiosis due to unknown species were reported in South Africa [29]. A 2018 case study described by Arsuaga et al. illustrates the difficulties of diagnosing babesiosis in the malaria-endemic areas of Cameroon and subsequently, Equatorial Guinea [179]. The complicated travel history of the patient in question coupled with the lack of available surveillance data on ticks and vertebrate reservoirs of *Babesia* species rendered it impossible for the authors to determine the definitive source of infection. Bloch and colleagues attribute the dearth of reported cases in Africa to a lack of surveillance data and to clinical and diagnostic overlap of *Babesia* with *Plasmodium* spp. in endemic areas [161]. In a pilot seroprevalence study, these authors examined seroreactivity among children in the Kilosa district of Tanzania. They concluded that *Babesia* may be present in the area, but that the potential for serological cross-reactivity and false positivity between *Babesia* and *Plasmodium* spp. impedes definitive conclusions about seroprevalence [161].

## 8. Babesiosis in Australia

A single autochthonous case of human babesiosis has been documented in Australia. Blood smear microscopy and molecular analysis revealed *B. microti* (United States type) as the infecting parasite [13]. A single imported case of babesiosis caused by *B. microti* infection also has been reported [180]. No evidence of *B. microti*-specific antibodies in 7000 blood donors and 29 clinically suspected babesiosis patients was detected in a serosurvey at multiple study sites in eastern Australia, leading the authors to conclude that transmission of *B. microti* is uncommon in this large region [181]. Babesiosis is a prevalent disease in cattle in Australia and is caused by *B. bigemina* and *B. bovis* [182]. Babesiosis is also prevalent in dogs where infecting species are *B. canis*, *B. vogeli*, and *B. gibsoni* [183]. Molecular studies demonstrating a tick-vector and reservoir-host for human *Babesia* spp. are lacking.

## 9. Conclusions

Human babesiosis is a worldwide emerging health problem that imposes a major disease burden, especially on the expanding older population and immunocompromised patients. Numerous studies indicate that the true number of *Babesia*-infected patients is markedly underestimated. As the infection continues to emerge, the number of affected individuals is likely to increase. Improved surveillance, as well as development of new antibiotics, supportive therapies, and a vaccine will all be important in limiting the impact of this disease.

## 10. Patents

Peter J. Krause is a co-applicant on a patent application entitled, “Enhanced Chemiluminescent enzyme-linked immunosorbent assay for detection of antibodies against *Babesia microti*”. The U.S. Provisional Patent Application No. 62/580,588 was filed on 2 November 2017. The patent is still under review. Dr. Krause has not received any funding for this patent.

## Data Availability

Not applicable.
