# Correction: Kumar et al. The Global Emergence of Human Babesiosis. *Pathogens* 2021, *10*, 1447

**DOI:** 10.3390/pathogens11050607

**Published:** 2022-05-23

**Authors:** Abhinav Kumar, Jane O’Bryan, Peter J. Krause

**Affiliations:** 1 Department of Epidemiology of Microbial Diseases, Yale School of Public Health and Yale School of Medicine, New Haven, CT 06510, USA; abhinav.kumar@yale.edu; 2 Department of Obstetrics, Gynecology & Reproductive Sciences, Yale School of Medicine, New Haven, CT 06510, USA; jane.obryan@yale.edu; 3 Frank H. Netter MD School of Medicine, Quinnipiac University, North Haven, CT 06473, USA

## Text Correction

There were two factual errors in the original publication of our manuscript [1] entitled, “The Global Emergence of Human Babesiosis”. *Babesia microti* is not transmitted transovarially. *Babesia crassa*-like agent has been found in *I. persulcatus* ticks in China rather than *I. scapularis* ticks. Several minor spelling and capitalization errors also were identified. 

The authors apologize for any inconvenience caused and state that the scientific conclusions are unaffected. This correction was approved by the Academic Editor. The original publication has also been updated.
