# Correction: Kumar et al. The Global Emergence of Human Babesiosis. *Pathogens* 2021, *10*, 1447

**DOI:** 10.3390/pathogens11080923

**Published:** 2022-08-17

**Authors:** Abhinav Kumar, Jane O’Bryan, Peter J. Krause

**Affiliations:** 1 Department of Epidemiology of Microbial Diseases, Yale School of Public Health and Yale School of Medicine, New Haven, CT 06510, USA; 2 Department of Obstetrics, Gynecology & Reproductive Sciences, Yale School of Medicine, New Haven, CT 06510, USA; 3 Frank H. Netter MD School of Medicine, Quinnipiac University, North Haven, CT 06473, USA

## Insert Patents

In the original publication [1], the patent application was not included. The correct patent application appears here:

## 10. Patents

Peter J. Krause is a co-applicant on a patent application entitled, “Enhanced Chemiluminescent enzyme-linked immunosorbent assay for detection of antibodies against *Babesia microti*”. The U.S. Provisional Patent Application No. 62/580,588 was filed on 2 November 2017. The patent is still under review. Dr. Krause has not received any funding for this patent. 

The authors apologize for any inconvenience caused and state that the scientific conclusions are unaffected. The original publication has also been updated.
